# Case Report: The application of novel imaging technologies in lower extremity peripheral artery disease: NIR-II imaging, OCTA, and LSFG

**DOI:** 10.3389/fcvm.2024.1460708

**Published:** 2024-09-18

**Authors:** Yijie Ning, Jie Hu, Haifeng Li, Chuanlong Lu, Zeyu Zhang, Sheng Yan, Peilu Shi, Tingting Gao, Heng Wang, Ruijing Zhang, Honglin Dong

**Affiliations:** ^1^ Department of Vascular Surgery, The Second Hospital of Shanxi Medical University, Taiyuan, China; ^2^ Key Laboratory of Big Data-Based Precision Medicine of Ministry of Industry and Information Technology, School of Engineering Medicine, Beihang University, Beijing, China; ^3^ Centre for Transplant and Renal Research, Westmead Institute for Medical Research, The University of Sydney, Sydney, NSW, Australia; ^4^ Department of Nephrology, The Second Hospital of Shanxi Medical University, Taiyuan, China

**Keywords:** peripheral artery disease, NIR-II imaging, optical coherence tomography, laser speckle flowgraphy, imaging technology

## Abstract

Lower extremity peripheral artery disease (PAD) is a growing global health problem. New methods to diagnose PAD have been explored in recent years. At present, the majority of imaging methods for PAD focus on the macrovascular blood flow, and the exploration of microcirculation and tissue perfusion of PAD remains largely insufficient. In this report, we applied three new imaging technologies, i.e., second near-infrared region (NIR-II, 900–1,880 nm wavelengths) imaging, optical coherence tomography angiography (OCTA), and laser speckle flowgraphy (LSFG), in a PAD patient with a healthy human subject as control. Our results showed that the PAD patient had poorer tissue perfusion than the control without observed adverse effects. Moreover, compared with the first near-infrared region (NIR-I, 700–900 nm wavelengths) imaging results, NIR-II imaging had a higher signal-to-background ratio and resolution than NIR-I imaging and detected microvessels that were not detected by NIR-I imaging. These observations suggested that NIR-II imaging, OCTA, and LSFG are potentially safe and effective methods for diagnosing PAD.

## Introduction

Peripheral artery disease (PAD) has been poorly defined at present (1). The American Heart Association (AHA) defines PAD as “atherosclerotic obstruction from the aortoiliac segments to the pedal arteries” (2), and we adopted the same definition in this report. PAD is the third leading cause of atherosclerotic morbidity, after coronary heart disease and stroke (3). The large number of patients and the poor prognosis of PAD have caused a heavy medical burden. PAD cases have risen each year since 1990 (4), and 238 million people were living with PAD in 2015 (1). Individuals with PAD have a higher risk of other cardiovascular diseases such as coronary heart disease, stroke, and abdominal aortic aneurysm (5, 6). Appropriate imaging technologies are key to preventing and treating PAD. The majority of imaging technologies for PAD detect vascular morphology on a macrovascular level. However, tissue oxygenation depends on the state of microcirculation (7). The AHA has noted PAD-related gaps, including new and non-invasive technologies to visualize peripheral perfusion (2), which emphasize the significance of evaluating microcirculation status in PAD patients.

We used three imaging technologies to evaluate microcirculation status in a PAD patient and a healthy human subject as control: second near-infrared region (NIR-II) imaging, optical coherence tomography angiography (OCTA), and laser speckle flowgraphy (LSFG) in this report. Previous studies of NIR imaging for diagnosing PAD have focused on the field of NIR-I (8). However, NIR-II imaging has shown lower scattering levels in tumor tissues and higher imaging resolution than NIR-I imaging (9). We applied NIR-II imaging to diagnose PAD for the first time and found that it had a higher signal-to-background ratio (SBR) and richer imaging details than NIR-I imaging. OCTA is a novel imaging method and has been widely used in ophthalmology and neuroscience research to observe retinal vessels and microvascular systems (10, 11). In this study, it was the first time that OCTA was applied to detect the microcirculatory system of the dorsal skin of the foot, to explore the potential of OCTA in diagnosing PAD. LSFG is a non-invasive detection technique for evaluating tissue perfusion, which is mainly used in retinal lesions (12). This study was also the first time that LSFG was used to detect the perfusion level of the dorsal skin of the foot .

This study is reported in accordance with the CARE guidelines (13).

## Case description

A 62-year-old male, with hypertension and type 2 diabetes mellitus, presented with intermittent claudication in both lower limbs for 2 years and worsening on the left side for 15 days. The patient was unable to walk more than 100 m at a time and was unable to complete the treadmill test. He did not experience rest pain or foot ulcers and did not receive any treatment for the above symptoms. Based on the findings from the medical history and examination, the patient was diagnosed with PAD, with Rutherford Category 3. Physical examination showed that the pulse of the popliteal artery, anterior tibial artery, and posterior tibial artery in the left lower limb was not palpated. The pulse of the posterior tibial artery in the right lower limb was not palpated. The ankle-brachial index (ABI) was 0.45 for the left and 0.81 for the right. Duplex ultrasound (DUS) showed severe stenosis of the left external iliac and superficial femoral arteries, multiple localized stenoses of the left popliteal artery, possible complete occlusion of the entire left posterior tibial artery, localized stenosis in the middle and lower segments of the right superficial femoral and popliteal arteries, and severe stenosis in the upper segment of the right posterior tibial artery. The results of computed tomography angiography (CTA) were consistent with DUS (Supplementary Figure S1).

A 52-year-old woman in good health was enrolled as a control. Physical examination showed that the pulsation of the femoral, popliteal, dorsalis pedis, and posterior tibial arteries in both lower limbs was palpated. DUS showed good blood flow filling in the arteries of both lower limbs. No significant abnormalities were observed in the spectral waveform or velocity of the blood flow. ABI was 1.08 for the left and 1.07 for the right limb.

## NIR imaging

NIR-II and NIR-I imaging were performed on both participants using a Full Spectrum Opening *in Vivo* Fluorescence Imaging System (DPM-IVFM-NIR-OF, Beijing Digital Precision Medicine Technology Co., Ltd., Beijing, China). An indent needle was placed in the basilic vein of the participants. The subject was in the supine position with both knee joints bent 90° and both feet put together. During NIR-II imaging, the selected filter was a 1,000 nm long pass. The power of the 808 nm wavelength laser emitter was adjusted to 10,000 mW with the aperture size adjusted to 2.0 and the exposure time set to 100 ms. The laser emitter was fixed at 20 cm vertical to the dorsal foot. Indocyanine green (ICG) solution (2.5 mg/ml, diluted with sterile water for injection) (Dandong Yichuang Pharmaceutical, Dandong, China) was administered intravenously via an indwelling needle. The dose of ICG injected was determined according to body weight: 0.1 mg/kg. The intensity of the fluorescence signal on both the dorsal feet was recorded within 5 min after the ICG solution was injected. The dorsal pedis region from the transverse tarsus joint to the distal metatarsal bone was selected as the region of interest (ROI). The ROI was analyzed to generate a time–intensity curve. The NIR-I imaging was performed 30 min after the NIR-II imaging. During NIR-I imaging, the xenon lamp was used as a laser emitter, with a xenon lamp filter of 750 nm band-pass and a NIR-I camera filter of 837 nm band-pass. The xenon lamp power was adjusted to 2,000 mW with the aperture size adjusted to 8.0 and the exposure time set to 200 ms. The laser emitter was fixed at 30 cm vertical to the dorsal foot. It is important to consider the impact of endogenous chromophores on NIR imaging based on ICG, as well as the influence of different skin tones on fluorescence intensity. Imaging parameters should be adjusted in response to changes in these factors (14).

**Figure 1:**
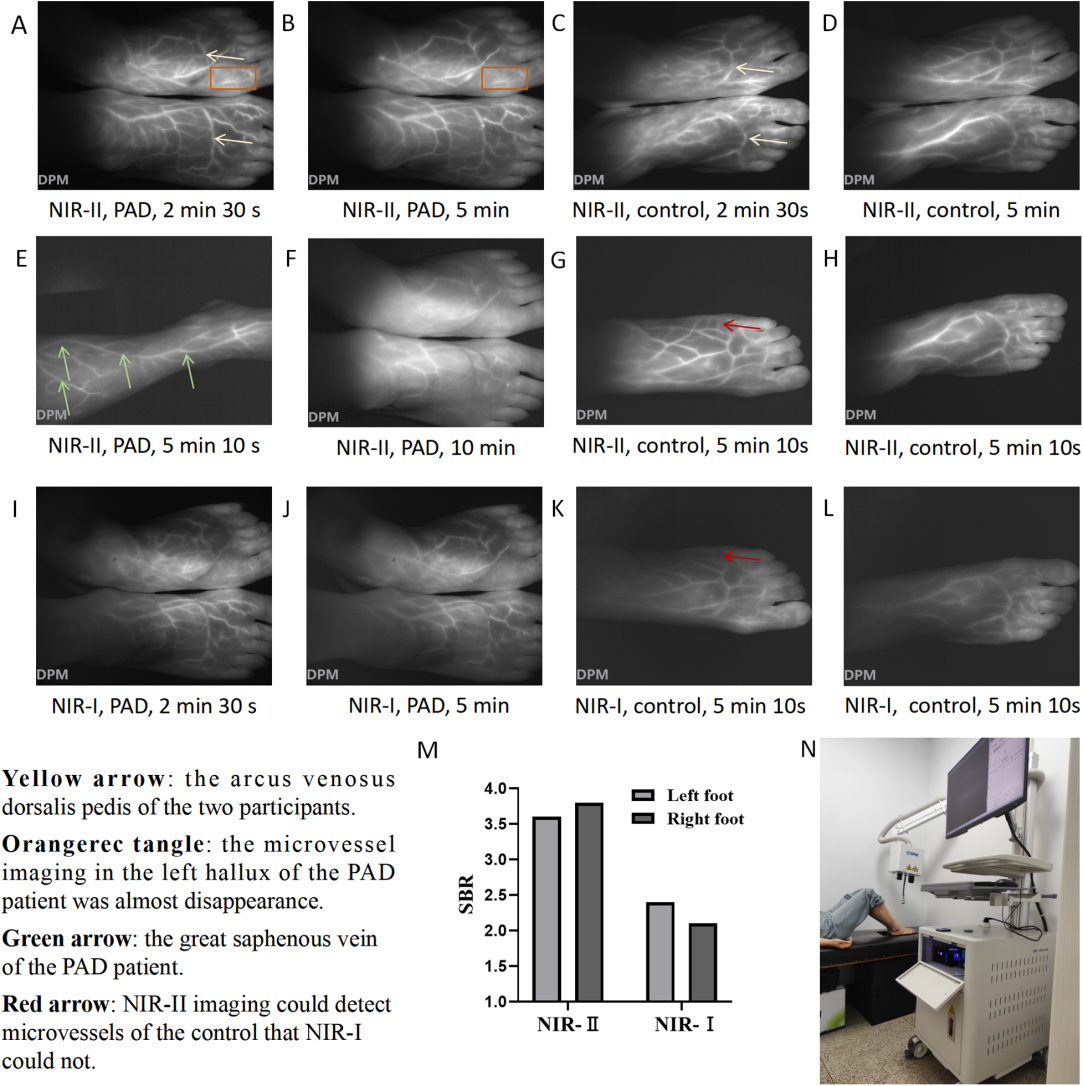
NIR imaging of the PAD patient and control. NIR-II images of the feet of the PAD patient at **(A)** 2 min 30 s and **(B)** 5 min. NIR-II images of the feet of the control at **(C)** 2 min 30 s and **(D)** 5 min. **(E)** NIR-II image of the right calf of the PAD patient at 5 min 10 s. **(F)** NIR-II image of the feet of the PAD patient at 10 min. NIR-II images of the **(G)** left foot and **(H)** right foot of the control at 5 min 10 s. NIR-I images of the feet of the PAD patient at **(I)** 2 min 30 s and **(J)** 5 min. NIR-I images of the **(K)** left foot and **(L)** right foot of the control at 5 min 10 s. **(M)** SBR of the left foot and right foot of the control under NIR-I and NIR-II imaging at 5 min 10 s. **(N)** The diagram of NIR imaging. Yellow arrow: the arcus venosus dorsalis pedis of the two participants. Orange rectangle: microvessel imaging in the left hallux of the PAD patient showed that the blood flow had almost disappeared. Green arrow: the great saphenous vein of the PAD patient. Red arrow: NIR-II imaging could detect microvessels of the control that NIR-I could not.

During NIR-II imaging, fluorescence images of the PAD patient (Figures 1A,B) and the control (Figures 1C,D) were captured at 2 min 30 s and 5 min, respectively. Microvessel imaging and venous imaging were observed. Fluorescence initially appeared (T start) in the dorsal pedis of the feet and then gradually emerged in the microcirculation. Fluorescence signals in both venous and microcirculatory imaging reflected the perfusion levels. The arcus venosus dorsalis pedis was clearly visible in both participants (yellow arrow). The microvessel imaging in the left hallux of the PAD patient showed that the blood flow had almost disappeared (orange rectangle), indicating severe ischemia here. At 5 min 10 s, we observed the fluorescence image of the right calf of the PAD patient (Figure 1E) and found that the great saphenous vein (green arrow) continued with the dorsalis pedis vein. At 10 min, we observed that the fluorescence signal of the PAD patient had decreased significantly (Figure 1F). At 5 min 10 s, we captured fluorescence images of the left foot (Figure 1G) and the right foot (Figure 1H) of the control, showing that a larger range of fluorescence signals in the dorsal foot could be observed by imaging with one foot. When imaging both feet, the laser toward the outermost part of the foot was frequently blocked by the slope of the dorsal foot, resulting in a lack of fluorescence signal. However, imaging with both feet was more consistent than imaging with one foot, by reducing the interference of operational errors. Furthermore, the injection of ICG solution was required only once, so imaging with both feet was chosen to record changes in fluorescence intensity. Imaging with one foot may be more suitable in certain circumstances, such as surgical operations. During NIR-I imaging, we observed that the resolution of imaging in the PAD patient at 2 min 30 s (Figure 1I) and 5 min (Figure 1J) was not as good as that of the resolution of NIR-II imaging at the corresponding times (Figures 1A,B). NIR-I fluorescence images of the left foot (Figure 1K) and the right foot (Figure 1L) of the control were captured at 5 min 10 s, respectively. We found that NIR-II imaging displayed microvessels that NIR-I imaging did not (red arrow). Furthermore, the SBR of NIR-II imaging (left foot: 3.6, right foot: 3.8) was higher than that of NIR-I imaging (left foot: 2.4, right foot: 2.1) within the ROI (Figure 1M). NIR-II and NIR-I images of one foot of the PAD patient at 5 min 10 s are shown in Supplementary Figures S2A,B. The SBR of NIR-II imaging was higher than that of NIR-I imaging within the ROI (2.9 vs. 1.8) (Supplementary Figure S2C). Furthermore, the SBR of the PAD patient was lower than the corresponding SBR of the control. The diagram of NIR imaging is shown in Figure 1N.

The time–intensity curve of NIR-II imaging was selected to quantitatively analyze the imaging results. Eleven parameters were used, including Imax, I start, I end, T start, Tmax, T 1/2, TR, Ingress, Ingress rate, Egress, and Egress rate. The definitions of the 11 parameters are detailed in Supplementary Table S1, and the parameter values of the two participants are shown in Table 1. According to the time–intensity curve of the PAD patient (Supplementary Figure S3A), the T start of the left foot (36.9 s) was significantly longer than that of the right foot (26.1 s). The Imax of the left foot (107.9) was lower than that of the right foot (137.9). The above data indicated that the perfusion level of the right foot was higher than that of the left foot in the PAD patient. Compared to the PAD patient, the Tmax of both feet was shorter in the control (left foot: 30.7 s, right foot: 42.3 s) (Supplementary Figure S3B). The ingress rate of both feet (left: 2.52, right: 1.83) in the control was higher than that of the PAD patient (left: 0.73, right: 0.76). The above data indicated that the perfusion level of both feet in the control was higher than that of the PAD patient, demonstrating the potential of NIR-II imaging in diagnosing PAD.

**Table 1:** Time–intensity curve parameters of the two participants.

Parameter	PAD patient		Control	
	Left foot	Right foot	Left foot	Right foot
Imax	107.9	137.9	162.1	160.9
I start	77.1	83.4	84.7	83.3
I end	89.5	98.5	113.1	113.3
T start	36.9	26.1	25.3	25.7
Tmax	42.3	74.7	30.7	42.3
T 1/2	14.0	20.9	18.4	19.0
TR	0.33	0.28	0.60	0.45
Ingress	30.8	54.5	77.4	77.6
Ingress rate	0.73	0.76	2.52	1.83
Egress	14.1	54.5	49.0	47.6
Egress rate	0.06	0.27	0.20	0.21

## OCTA

OCTA imaging was performed in both participants using a Monitoring System of Vascular Microcirculation *in vivo* (Micro-VCC, Optoprobe Science Ltd, Pontypridd, UK). The imaging depth was 0–3 mm. The field of view scanned by OCTA was 9 mm × 9 mm. We selected the area between the first and second metatarsal heads of the dorsal foot as the ROI. The detection probe was adjusted to closely adhere to the skin of the ROI. The detection time of each foot was approximately 60 s without the injection of a developer. The diagram of OCTA imaging is shown in Supplementary Figure S4A. Using the analysis software Pyoct (version 8.0), we selected a depth of 300–1,000 um from the skin to analyze the results, set the analysis threshold of the image to 0.5, and then obtained a Gray Scale Image, Pseudocolor Image, Skeleton Image, and four kinds of heat maps: Area Heat Map, Complexity Heat Map, Diameter Heat Map, and Skeleton Heat Map. The images of each foot were quantitatively analyzed so that the average area, average complexity, average diameter, and average skeleton density could be obtained. In addition, to explore the influence of different imaging depths on the imaging effect of OCTA, we observed the right foot of the control at 0–200, 200–300, and 1,000–1,200 μm imaging depths, respectively.

**Figure 2:**
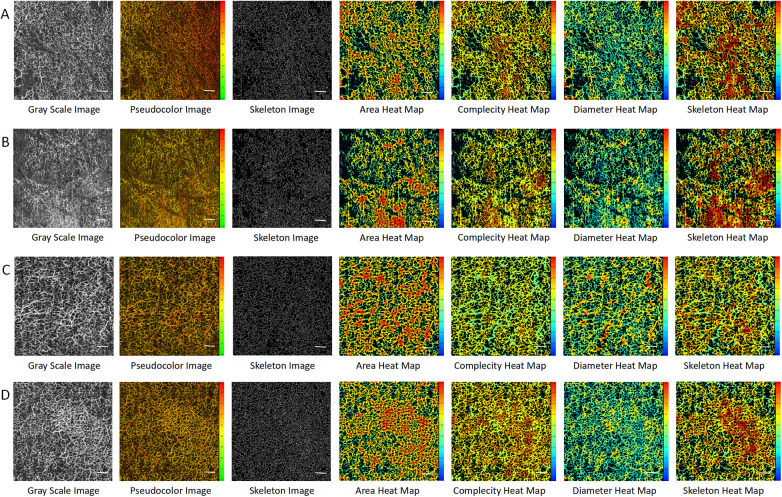
OCTA images in the PAD patient and the control at imaging depths of 300–1,000 μm. **(A)** The left foot of the PAD patient. **(B)** The right foot of the PAD patient. **(C)** The left foot of the control. **(D)** The right foot of the control. Scale bar = 100 um.

At an imaging depth of 300–1,000 um, by comparing the images of the left foot (Figure 2A) and right foot (Figure 2B) of the PAD patient with those of the left foot (Figure 2C) and right foot (Figure 2D) of the control, we found that the microvessels in the OCTA images of the control were more densely distributed and larger in diameter. This was consistent with the OCTA quantification results (Supplementary Table S2). The average areas of the left and right feet of the PAD patient were 0.447 and 0.430, respectively, compared to 0.471 and 0.497 in the control. The average diameters of the left and right feet in the PAD patient were 34.157 and 32.790 μm, compared to 41.626 and 37.517 μm in the control. The average area and average diameter of microvessels in the feet of the control were higher than those of the PAD patient. We speculated that the average area and average diameter have the potential to be the key parameters in diagnosing PAD.

At an imaging depth of 0–200 μm, almost no microvessels could be observed in the OCTA image of the right foot of the control, while skin texture and hair could be observed (Supplementary Figure S4B). At an imaging depth of 200–300 μm, emerging microvessels in the papillary layer could be observed, but the microvascular profile could not be observed (Supplementary Figure S4C). At an imaging depth of 1,000–1,200 um, the connective tissue of the hypodermis could be observed, but it was difficult to observe the complete microvascular structure (Supplementary Figure S4D). Therefore, the appropriate depth for observing the microcirculatory system of the dorsal foot with OCTA was 300–1,000 μm.

## LSFG

LSFG was performed on both participants. The imaging device used was the Laser Speckle Imaging System (RFILSI ZW, RWD Life Science, Shenzhen, China) with an imaging depth of 0–1 mm. The perfusion image and the intensity image were captured at the same processing mode (Sliding mode), spatial filtering constant (3 s), temporal filtering constant (250 frames), and frame rate (40 fps). During the imaging, the participant sat on the examination chair, and the knee joint of the examination side was bent at 90° so that the foot could be flat on the black cardboard on the examination plane. The position of the speckle host was adjusted so that the dorsal foot was fully exposed in the center of the field of vision. The imaging diagram is shown in Supplementary Figure S5. The working distance was adjusted to 25 cm. The dorsal foot from the transverse tarsal joint to the distal metatarsal bone was selected as the ROI, and the change in perfusion volume in the ROI was observed. When the perfusion volume was relatively stable, the measurement was performed for 5 s. RFLSI Analysis software was used to obtain perfusion-related parameters (maximum perfusion, minimum perfusion, mean perfusion, and standard deviation) and intensity-related parameters (maximum intensity, minimum intensity, mean intensity, and standard deviation).

**Figure 3:**
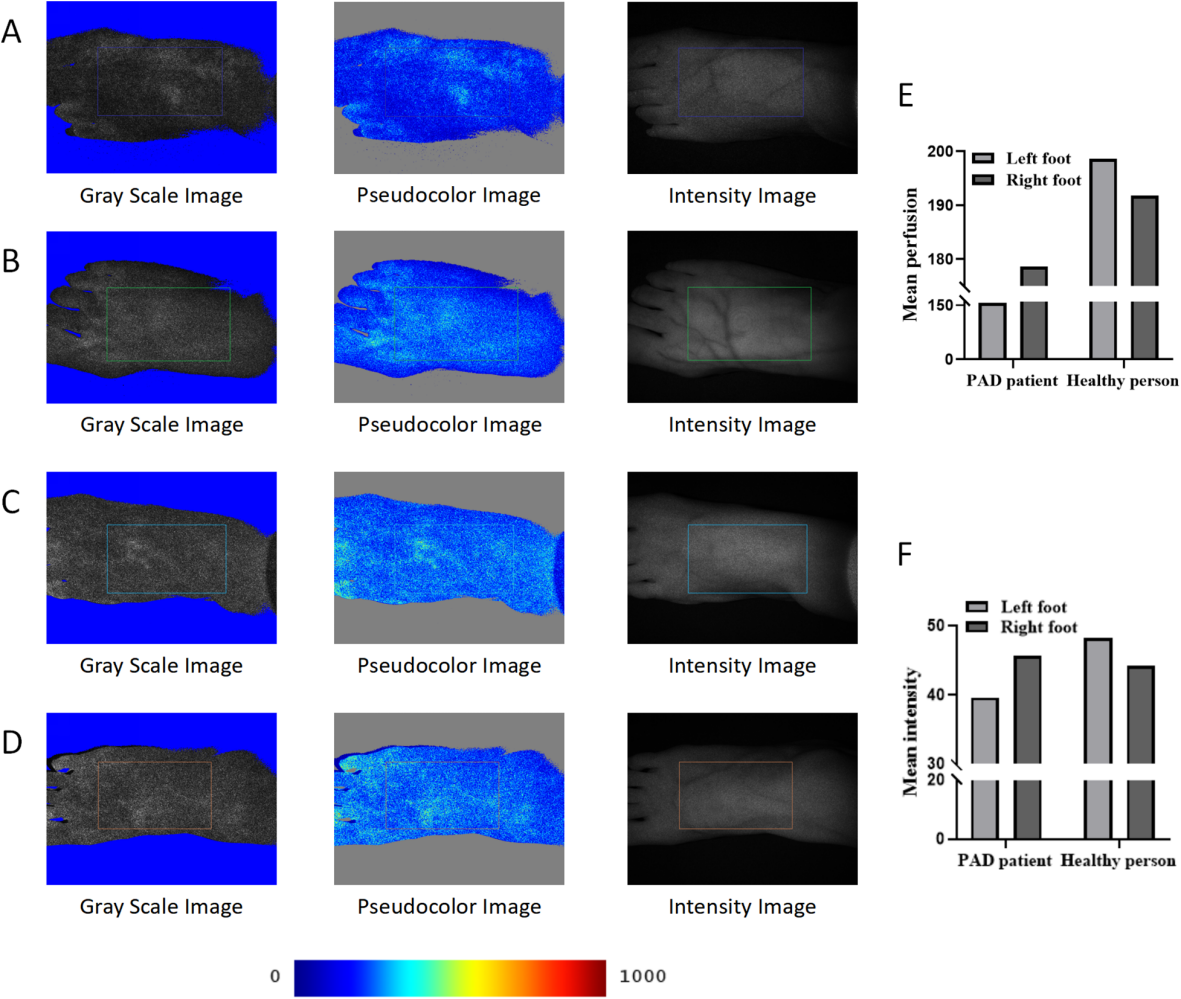
LSFG of the PAD patient and the control. **(A)** LSFG images of the left foot of the PAD patient. **(B)** LSFG images of the right foot of the PAD patient. **(C)** LSFG images of the left foot of the control. **(D)** LSFG images of the right foot of the control. **(E)** Mean perfusion of each foot of the two participants. **(F)** Mean intensity of each foot of the two participants. The color bar represents perfusion levels, from blue (low) to red (high). ROIs are framed with rectangles.

By comparing the gray images and pseudocolor images of the two participants, we found that the perfusion level and intensity of the left foot (Figure 3A) of the PAD patient were lower than those of the right foot (Figure 3B). The perfusion levels of the left foot (Figure 3C) and the right foot (Figure 3D) of the control were almost the same, and both were higher than those of the PAD patient. The perfusion volume results were consistent with the quantitative results (Supplementary Table S3). The mean perfusion of the left foot of the PAD patient was 156.44, which was lower than 178.66 in the right foot. The mean perfusion of the left foot and the right foot of the control was 198.68 and 191.80, respectively (Figure 3E), higher than that of the PAD patient, reflecting the consistency between the perfusion volume of the dorsal foot and the degree of PAD lesions. However, the mean intensity of the right foot of the control was 44.20, which was lower than 45.67 of the PAD patient (Figure 3F). This may be due to the different smoothness of the skin on the dorsal foot between the two participants and the intensity measurement being more easily affected by the smoothness of the skin.

## Discussion

When PAD occurs, in addition to the decrease in the patency of the large arteries, the microcirculatory system will also be affected accordingly. In the presence of atherosclerosis, the microcirculation function changes occur earlier than the macrovascular status (15). In intermittent claudication, decreased blood flow in the microcirculation is an important pathophysiological change (16). Tissue oxygenation depends on the state of microcirculation, and microcirculation-related diagnostic techniques can help us identify areas of poor perfusion, which has the potential to guide the surgery and predict the prognosis (7, 17). Therefore, diagnostic techniques for microcirculatory systems are as important as macrovascular diagnostic techniques for PAD. However, the diagnostic technology for tissue perfusion still needs to be improved at present. Although transcutaneous oxygen pressure (TcPO_2_) is the most commonly used diagnostic technology for tissue perfusion in clinical practice, it has shortcomings such as being time-consuming and having a small detection range (18). We report the clinical application of NIR-II imaging, OCTA, and LSFG, showing that these three novel technologies have the potential to detect microcirculatory structure and tissue perfusion levels in PAD.

Based on the imaging results of the two participants, NIR-II imaging had a higher resolution and SBR than NIR-I imaging in diagnosing PAD. NIR-II imaging could visualize the microvessels that were not visualized by NIR-I imaging. Furthermore, NIR-II imaging could visualize the poor perfusion area of the dorsal foot. Due to the differences in imaging conditions between NIR-II and NIR-I imaging (including imaging distance, imaging sequence, exposure time, and laser light source) and the limited sample size of this study, it is not rigorous to conclude that NIR-II imaging is better than NIR-I imaging. Future studies with larger sample sizes are needed to explore the effects of both types of imaging. The parameters of the time–intensity curve were significantly different between the control and the PAD patient, indicating that NIR-II imaging had a good ability to distinguish PAD. NIR-II imaging could visualize the microvessels of the dorsal foot in a non-contact manner in real-time, indicating its potential for intraoperative applications. NIR imaging has a wide range of applications (19). Previous studies have reported that NIR imaging could assess the perfusion status in patients with chronic limb ischemia vs. control patients (20). Meanwhile, NIR imaging could predict clinical outcomes after revascularization for lower extremity arterial disease (21). However, NIR-II imaging cannot determine the specific lesion site in lower extremity arteries, and each part of the time–intensity curve may contain information about the lesion site or the degree of the lesion, which needs to be explored with larger sample size studies. In addition, sex-related differences need to be considered in the evaluation of microcirculation, which may be due to differences in blood pressure or hormones (22). The prevalence of PAD also showed a sex difference, with men having a higher prevalence (23).

OCTA is widely used in ophthalmology and neuroscience research (9, 10), and has also been reported to have been applied to skin diseases (24). This study is the first to report the application of OCTA in diagnosing PAD. We found that the average area and average diameter were higher in the control than in the PAD patient, suggesting that the two parameters might be important for diagnosing PAD. The advantage of OCTA is that the microcirculation structures at different depths can be observed. Results showed that 300–1,000 nm was the appropriate depth to observe microcirculation in PAD patients. However, in OCTA imaging, the results can be interfered with by slight movement from the patient, leading to re-examination, so the skin in the ROI must be attached to the probe tightly.

LSFG has been used to observe the plantar skin for diagnosing PAD in previous reports (25). Since the imaging depth of the device is only 1 mm, the dorsal foot skin was selected as the ROI in this study. LSFG can assess the perfusion level easily and quickly, without touching the skin. The average perfusion of the two participants showed a significant difference in grayscale. The intensity-related parameters may be affected by the reflection of the skin, and their accuracy is not as good as that of the perfusion-related parameters. In the future, we will continue to pay close attention to this issue and further explore the diagnostic efficacy of intensity-related parameters.

## Conclusions

In summary, this study reported the clinical application of NIR-II imaging, OCTA, and LSFG for PAD. They showed differences in perfusion levels between the PAD patient and the control without adverse effects, suggesting promising potential for clinical application. Further larger-sample studies are warranted to determine the diagnostic thresholds for these three novel diagnostic technologies.

## Data Availability

The original contributions presented in the study are included in the article/Supplementary Material, further inquiries can be directed to the corresponding authors.
