# Supplementary material for: Case Report: The application of novel imaging technologies in lower extremity peripheral artery disease: NIR-II imaging, OCTA, and LSFG
Source: Front Cardiovasc Med. 2024 Sep 18;11:1460708. doi: 10.3389/fcvm.2024.1460708 (PMC11444973; doi:10.3389/fcvm.2024.1460708)
Supplement: Supplementary file 1 [file Datasheet1.docx]

Supplementary Material

**Supplementary Figures**

**
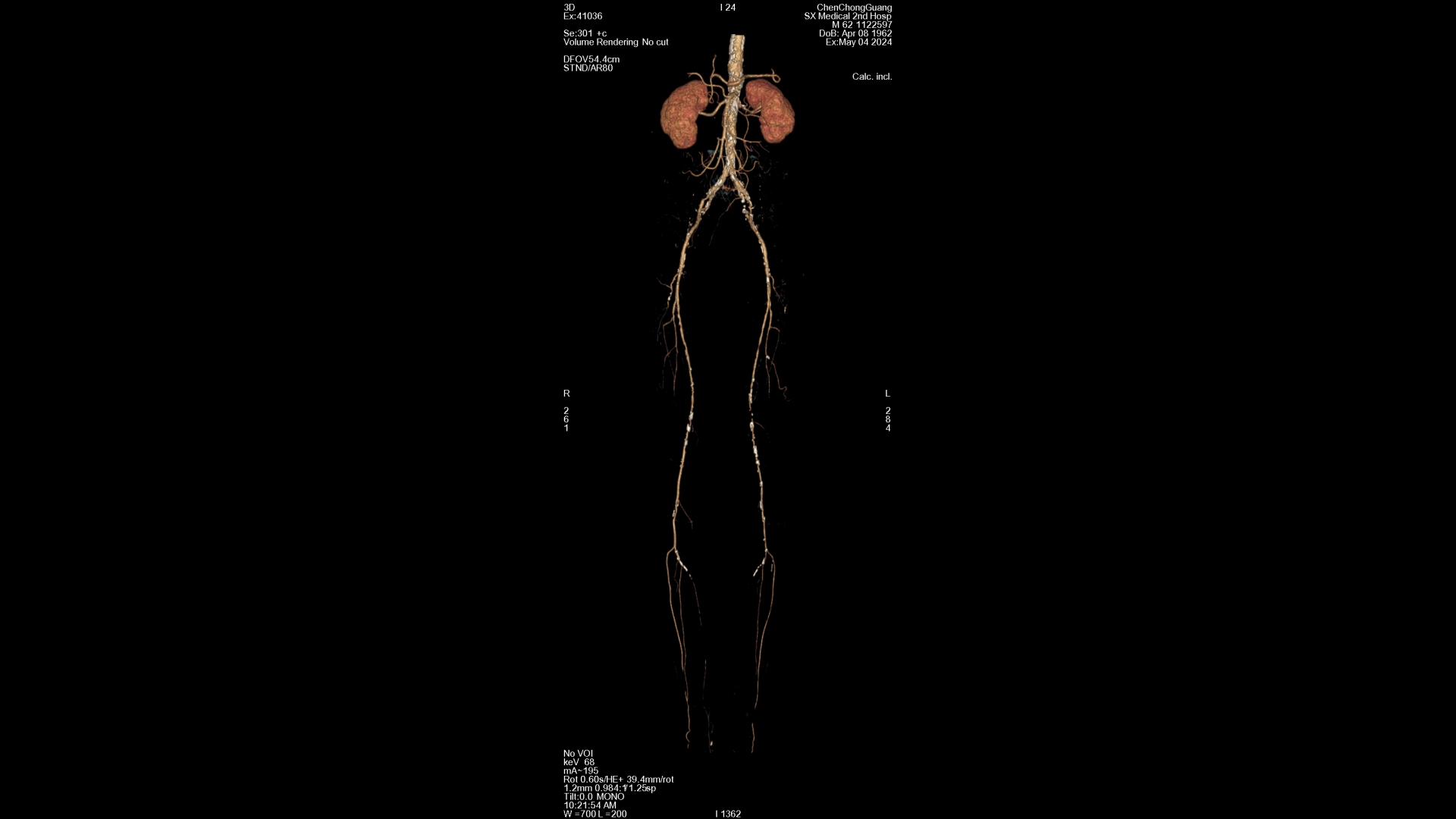
**

**Supplementary Figure 1.** CTA imaging of the PAD patient.

**
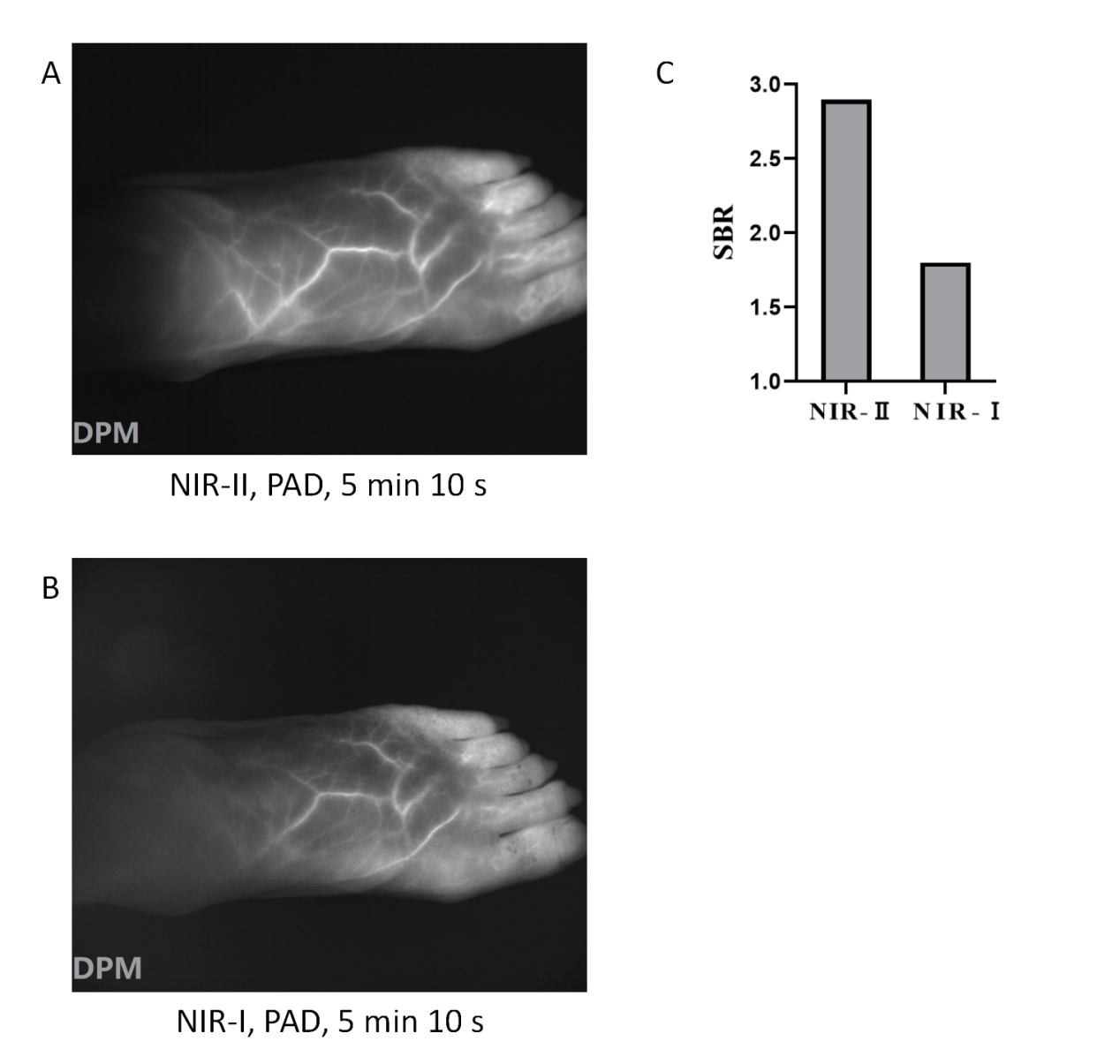
**

**Supplementary Figure 2.** NIR images with one foot and the SBR of the PAD patient at 5 min 10 s. (A) NIR-II image and (B) NIR-I image with one foot of the PAD patient at 5 min 10 s. (C) The signal-to-background ratio (SBR) with one foot of the PAD patient under NIR-I and NIR-II imaging at 5 min 10 s.


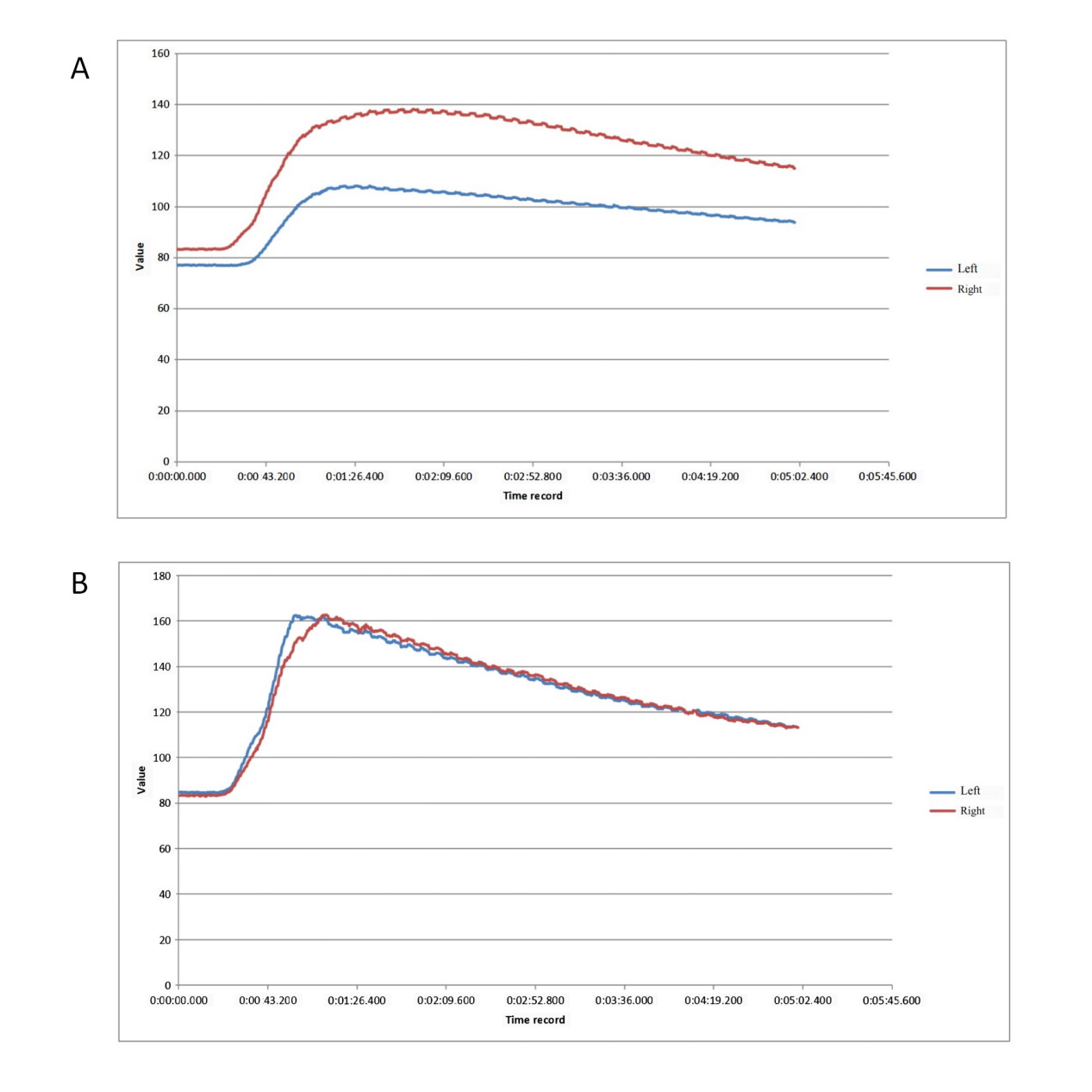
 **Supplementary Figure 3.** Time-intensity curves of the PAD patient and the control shown by NIR-II imaging. (A) PAD patient. (B) Control.

**
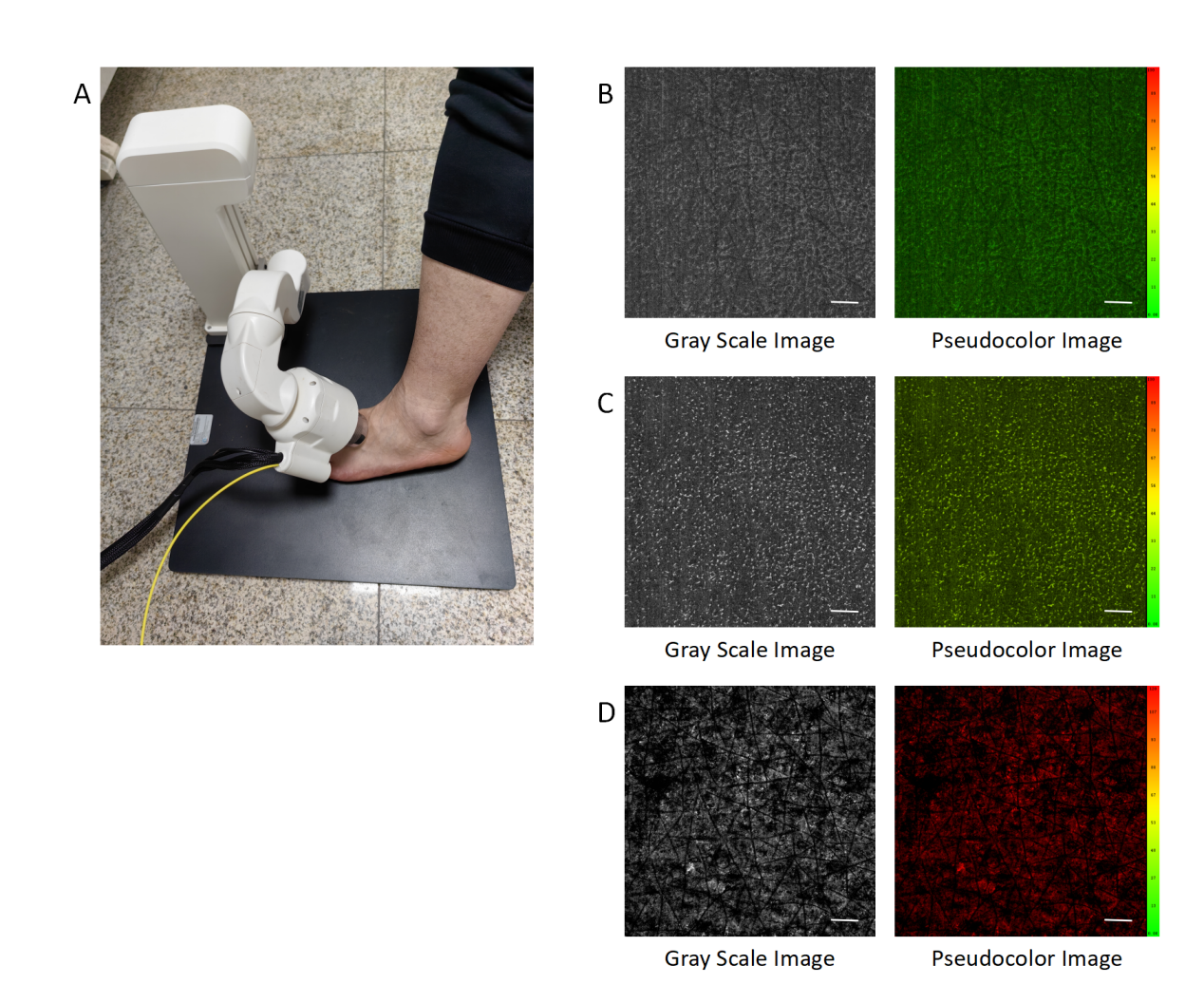
Supplementary Figure 4.** The scene of OCTA imaging and the OCTA images (gray scale image and pseudocolor image) of the right foot of the control at different levels. (A)The scene of OCTA imaging. (B) 0-200 um. (C) 200-300 um. (D) 1000-1200 um. Scale bar = 100 um.


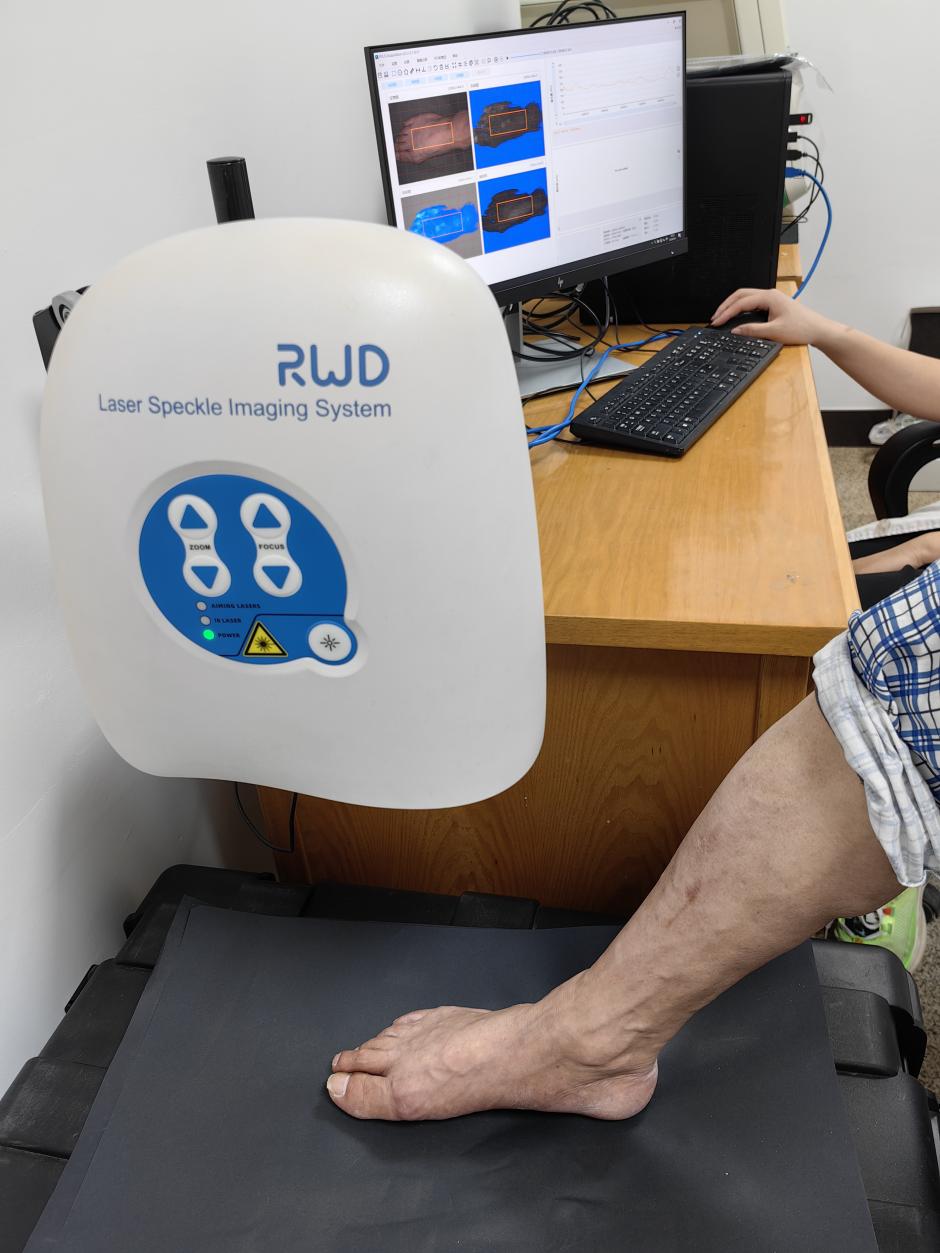


**Supplementary Figure 5.** The scene of Speckle imaging.

**Supplementary Tables**

**Supplementary Table 1.** Interpretation of time-intensity curve parameters

| Parameter | Definition | Equivalent |
| --- | --- | --- |
| Imax | Maximum fluorescence intensity | - |
| I start | Initial fluorescence intensity | - |
| I end | the final intensity at 300 s | - |
| T start | Time from injection of ICG solution to initial fluorescence | - |
| Tmax | Time from initial fluorescence to maximum fluorescence intensity | - |
| T 1/2 | Time from initial fluorescence to half of the maximum fluorescence intensity | - |
| TR | Time ratio (T1/2/Tmax) | - |
| Ingress | Absolute difference between Initial intensity and its maximum value | Imax - I start |
| Ingress rate | Rate of increase of fluorescence from Initial fluorescence to maximum value | (Imax - I start)/Tmax |
| Engress | Absolute difference between maximum intensity and the final intensity | Imax - I end |
| Engress rate | Rate of decrease of fluorescence from maximum value to the final intensity value | (Imax - I end)/(300 s - T start - Tmax) |

**Supplementary Table 2.** OCTA parameters of the two participants.

| Parameter | PAD patient | | Control | |
| --- | --- | --- | --- | --- |
|  | Left foot | Right foot | Left foot | Right foot |
| Average area | 0.447 | 0.430 | 0.471 | 0.497 |
| Average complexity | 0.637 | 0.631 | 0.606 | 0.638 |
| Average diameter (um) | 34.157 | 32.790 | 41.626 | 37.519 |
| Average skeleton density | 0.136 | 0.137 | 0.124 | 0.136 |

**Supplementary Table 3**. LSFG parameters of the two participants.

| Image type | Parameter | PAD patient | | Control | |
| --- | --- | --- | --- | --- | --- |
|  |  | Left foot | Right foot | Left foot | Right foot |
| Gray Scale Image | Maximum perfusion | 167.68 | 185.62 | 204.66 | 194.76 |
|  | Minimum perfusion | 149.32 | 171.02 | 193.21 | 189.08 |
|  | Mean perfusion | 156.44 | 178.66 | 198.68 | 191.80 |
|  | Standard deviation | 4.92 | 3.87 | 3.02 | 1.08 |
| Intensity Image | Maximum intensity | 39.75 | 45.87 | 48.43 | 44.27 |
|  | Minimum intensity | 39.53 | 45.54 | 48.14 | 44.17 |
|  | Mean intensity | 39.65 | 45.67 | 48.30 | 44.20 |
|  | Standard deviation | 0.05 | 0.06 | 0.06 | 0.02 |
